# Linguistic realizations of personality and emotional polarity in Tan Twan Eng’s *The Garden of Evening Mists*: A systemic functional discourse analysis

**DOI:** 10.1371/journal.pone.0354255

**Published:** 2026-07-24

**Authors:** Mi Chen

**Affiliations:** School of Foreign Languages, Fuzhou University of International Studies and Trade, Fuzhou, Fujian, China; Chengdu Normal University, CHINA

## Abstract

This study examines how personality and emotion are linguistically realized in Tan Twan Eng’s *The Garden of Evening Mists*, drawing on the Big Five Model of Personality (BFM) and Systemic Functional Linguistics (SFL) to construct an integrated theoretical-qualitative framework for the analysis of character language in literary narrative. Emotional polarity is understood as the systematic orientation of affective meaning, positive, neutral, or negative, realized through lexicogrammatical choices at the interpersonal, ideational, and textual levels of language. Through close discourse analysis of selected passages, the study traces how patterns of modality, process type, and cohesion correspond to shifts in emotional polarity across three narrative phases: trauma recollection, apprenticeship in Yugiri, and reflective closure. The findings indicate that emotional polarity in the novel can be read as a form of psychological adaptation rather than static sentiment: negative polarity, realized through obligation modals and syntactic compression, reflects a high-Conscientiousness, high-Neuroticism configuration associated with trauma-related self-regulation; neutral polarity, marked by evaluative balance and descriptive cohesion, signals stabilized Conscientiousness and emerging Openness; positive polarity, expressed through relational processes and permissive modality, corresponds to increased Openness and diminished Neuroticism. The interpersonal dimensions of Agreeableness and Extraversion are realized at lower density but trace parallel arcs across the three phases, with Agreeableness providing the linguistic site at which the novel’s central thematic question of inherited hatred is settled. By integrating Big Five personality theory with systemic-functional analysis, the study offers a theoretically grounded and interpretively transparent framework for investigating how emotional language encodes personality transformation in narrative discourse. The findings contribute to ongoing dialogues between personality psychology, functional linguistics, and literary stylistics and illustrate how this framework can be applied to postcolonial narrative fiction.

## 1. Introduction

The intersection of literary studies and emotion research has attracted increasing scholarly attention, reflecting a broader interdisciplinary turn toward affective dimensions of narrative [1]. Research tracing personality structures in literary texts across historical periods suggests that fictional narratives encode implicit models of personality that both reflect and shape cultural understandings of individual differences [2,3]. The study of character personality in literary texts has developed along two main lines. The first approach focuses on the qualitative interpretation of literature, in which researchers analyse a character’s psychological traits through close reading and narrative interpretation. Based on the critic’s humanistic insight and interpretive experience, personality features are inferred from textual evidence such as description, dialogue, and internal monologue, and then generalised across an author’s works at the macro level [4]. The second approach originates from linguistic and corpus-based analysis, in which character personality and emotion are examined through linguistic data. By identifying high-frequency words, dialogue structures, and pragmatic markers, researchers can model a character’s emotional tendencies and cognitive style. This linguistic perspective aligns with the principles of Systemic Functional Linguistics (SFL), which connects grammatical and lexical choices to meaning-making in social and psychological contexts [5,6].

Although psychoanalytic readings have provided valuable interpretive insight, they often depend on subjective experience and lack systematic linguistic grounding [7]. The emergence of the Big Five Model of Personality (BFM) introduced measurable dimensions of personality, namely openness, conscientiousness, extraversion, agreeableness, and neuroticism, offering a structured way to describe individual differences [8]. Parallel developments in corpus-based and psycholinguistic approaches to literary analysis have enabled more systematic examination of emotional and stylistic variation in texts [9,10]. In this context, Emotional Polarity Analysis (EPA) has become an important lens for identifying affective patterns in narrative discourse, with scholars increasingly attending to how positive, neutral, and negative emotional orientations are realized through specific lexicogrammatical choices rather than surface sentiment labels [11].

From a psycholinguistic perspective, personality and emotion are not independent constructs but mutually constitutive dimensions of psychological experience realized through language. Stable personality traits shape the affective orientation with which individuals interpret and respond to events, while emotional states, when consistently patterned across discourse contexts, provide observable evidence of underlying personality dispositions [12,13]. In literary narrative, this relationship is particularly salient: a character’s habitual linguistic choices, such as the modals they favour, the processes they enact, and the evaluative stance they adopt, encode both immediate emotional response and enduring psychological tendency. Systemic Functional Linguistics provides the analytical bridge between these two levels, treating interpersonal meaning as the site where affect and personality simultaneously find expression [6]. To analyse one without the other, in this context, would be to sacrifice the psychological coherence that gives literary character its depth and consistency.

Despite these advances, traditional EPA methods often fail to capture the subtle dependencies and figurative meanings that shape emotion in literary language. They tend to rely on surface lexical features while overlooking contextual and grammatical nuance. Moreover, existing scholarship has approached this terrain through complementary but disconnected paths. Computational personality detection studies [14,15] have established robust BFM-language correspondences but have done so on social media and self-report corpora, not on extended literary narratives where personality is constructed through retrospective voice rather than spontaneous self-report. Literary stylistics in the SFL tradition [16]; Matthiessen and Yousefi [6] have analyzed emotional and evaluative meaning in fiction with grammatical precision, but have rarely connected these analyses to a structured psychological framework such as the BFM. Recent work has begun to bridge this gap: Du and Bauditz [2,3] demonstrate BFM-coherent linguistic patterns in literary texts, yet the corpus base of this emerging literature remains anchored in canonical Anglo-American fiction. Postcolonial Anglophone narratives, and Malaysian English literature in particular, have not yet been brought into this analytical conversation, despite offering precisely the kind of layered psychological discourse for which BFM-SFL analysis is designed. The present study addresses this opening by applying the integrated framework to *The Garden of Evening Mists*, treating the novel as a demonstrative case for what such analysis can yield when extended to a postcolonial Asian Anglophone literary context.

To address these gaps, this study proposes a psycholinguistically informed approach to literary psychology, integrating the Big Five Model of Personality (BFM) and Systemic Functional Linguistics (SFL). The analysis focuses on *The Garden of Evening Mists* by Tan Twan Eng, a novel that traces Teoh Yun Ling’s journey from trauma and repression to reconciliation through the intertwined processes of memory, language, and silence.

The selection of this novel is motivated by several considerations. Its first-person retrospective narration creates a sustained convergence between the narrator’s and the protagonist’s voices, providing an analytically stable site for the examination of interpersonal meaning and emotional stance. The novel’s three-phase psychological arc, moving from trauma and repression, through aesthetic discipline, to reconciliation and acceptance, offers a structurally coherent basis for tracing personality transformation across the narrative. Furthermore, the text’s characteristic restraint, in which emotion is conveyed through silence, modal nuance, and sensory precision rather than overt affective declaration, makes it particularly responsive to the kind of grammatical and discoursal analysis that SFL enables.

As a postcolonial narrative engaging questions of memory, identity, and moral selfhood, *The Garden of Evening Mists* occupies a significant position in Malaysian English literary studies, extending the application of psycholinguistic frameworks to underrepresented literary traditions and addressing a gap in the field [9,11,17,18]. The present study is explicitly framed as a single-text demonstrative analysis: its aim is not statistical generalizability but the theoretically grounded illustration of how the proposed BFM-SFL framework operates as an interpretive tool for personality-oriented literary analysis. By mapping emotional polarity and linguistic patterns onto personality dimensions, the study investigates how psychological transformation is realized through language.

This interdisciplinary framework combines linguistic description and psychological theory to provide an interpretively grounded account of emotion and personality in narrative discourse. Rather than reducing character psychology to surface lexical features, the SFL-based close reading adopted in this study attends to the grammatical and discoursal resources through which emotion and identity are constructed across the narrative arc. In doing so, the study contributes to ongoing dialogues between psycholinguistics, personality theory, and literary analysis, offering a theoretically coherent and analytically transparent framework for investigating how language encodes the dynamics of emotion and selfhood in postcolonial narrative fiction.

## 2. Theoretical basis and model establishment

### 2.1. Theoretical Basis

The interdisciplinary study of personality and emotion in literature brings together perspectives from psychology, linguistics, and psycholinguistics. This research draws upon two complementary frameworks: the Big Five Model (BFM) of Personality and Systemic Functional Linguistics (SFL), informed by psycholinguistic theory linking language use to personality expression and emotional regulation, to establish a conceptual foundation for understanding how language encodes psychological traits and emotional states in narrative discourse.

#### 2.1.1. Personality theory.

Personality psychology provides the structural basis for analysing individual differences in both real and fictional contexts. The Big Five Model (BFM) identifies five core personality dimensions, namely openness, conscientiousness, extraversion, agreeableness, and neuroticism, that describe consistent psychological patterns across individuals [8]. Each dimension captures a different aspect of emotional and behavioural regulation: openness reflects curiosity and imagination; conscientiousness, self-control and organisation; extraversion, social engagement; agreeableness, empathy and cooperation; and neuroticism, emotional instability [15]. To illustrate how linguistic features correspond to these psychological tendencies, Table 1 summarizes the five dimensions of the BFM alongside their typical linguistic manifestations.

**Table 1:** Dimensions of the Big Five Model (BFM) of Personality The table illustrates how linguistic features may reflect corresponding psychological tendencies within the BFM framework. “Evaluative lexis” refers to lexical items encoding value judgements along attitudinal axes, corresponding to the Attitude system in Appraisal Theory [25]. “Affective intensity” refers to the scalar amplification or mitigation of emotional meaning, corresponding to the Graduation system within the same framework [25]. These two categories are analytically distinct, though they may co-occur in the same clause. Linguistic indicator sets are representative rather than exhaustive.

Dimension	Psychological Tendency	Description	Typical Linguistic Indicators
**Openness**	Imagination, aesthetic sensitivity	Cognitive flexibility, creativity, curiosity	Abstract nouns; perceptual adjectives; mental process verbs (*see*, *discover*, *wonder*)
**Conscientiousness**	Organization, self-discipline	Goal orientation, moral restraint, precision	Modal verbs of obligation (*must*, *cannot*, *will not*); precise action verbs; negation structures
**Extraversion**	Sociability, assertiveness	Expressive interaction, enthusiasm, social engagement	First-person pronouns; high affective intensity; declarative mood; positive appraisal lexis
**Agreeableness**	Compassion, cooperation	Altruism, interpersonal warmth, empathy	Positive politeness markers; positive appraisal lexis. Negative politeness strategies may also index Conscientiousness or Neuroticism [19]
**Neuroticism**	Emotional sensitivity, anxiety	Stress tendency, vulnerability, emotional instability	Negative adjectives; hedging modals (*might*, *perhaps*); affective lexis of withdrawal and pain

The correspondence between BFM dimensions and their linguistic realizations has been empirically established across a range of text-based personality studies. Research on self-narrative language has demonstrated that Conscientiousness is reliably indexed by obligation modals, precise action verbs, and structured syntactic patterns reflecting goal orientation and self-discipline [20]. Openness and Extraversion, by contrast, are associated with perceptual adjectives, first-person pronouns, and markers of high affective intensity, reflecting the imaginative engagement and social expressiveness characteristic of these dimensions [21]. These empirically grounded correspondences inform the linguistic indicators summarized in Table 1 and provide the evidential basis for the BFM–SFL mapping developed in the analytical framework.

The Big Five Model is adopted in the present study for several reasons. First, it has accumulated the most extensive empirical validation in personality psychology and remains the dominant framework in both cross-cultural and text-based personality research [14,15]. Second, its five dimensions, particularly Conscientiousness, Neuroticism, and Openness, map directly onto the patterns of emotional regulation, moral tension, and psychological transformation that characterize Yun Ling’s narrative voice in *The Garden of Evening Mists*. Third, the BFM has been productively applied in text-based personality analysis, including the systematic examination of fictional characters across different literary periods, providing an established basis for linking linguistic features to psychological dimensions. [3,14]. While more recent models offer additional dimensions that may prove valuable in future cross-cultural literary research, the present study prioritizes the BFM’s analytical clarity and its proven applicability to language-based personality inference within a single-text qualitative framework.

Building on this descriptive model, McAdams proposed a three-tier personality theory that introduces a developmental and narrative dimension [7]. Personality operates at three levels: dispositional traits, characteristic adaptations, and life narratives. The first two represent stable tendencies and contextual motivations, while the third, narrative identity, captures the individual’s self-story constructed through memory and meaning-making. This framework is particularly valuable for literary analysis, where language, reflection, and memory serve as the primary means through which psychological evolution becomes visible.

Recent research has extended the BFM toward cross-linguistic and text-based domains. Soto and John revised the Big Five Inventory (BFI-2) to account for cultural and linguistic variability [15]. Park and his colleagues demonstrated that natural language use can predict personality profiles, confirming that linguistic cues reliably signal personality dimensions [14]. More recently, scholars have extended BFM-based analysis to literary texts across historical periods, demonstrating that fictional narratives encode implicit models of personality that reflect broader cultural understandings of individual differences [2,3].

**Fig 1:**
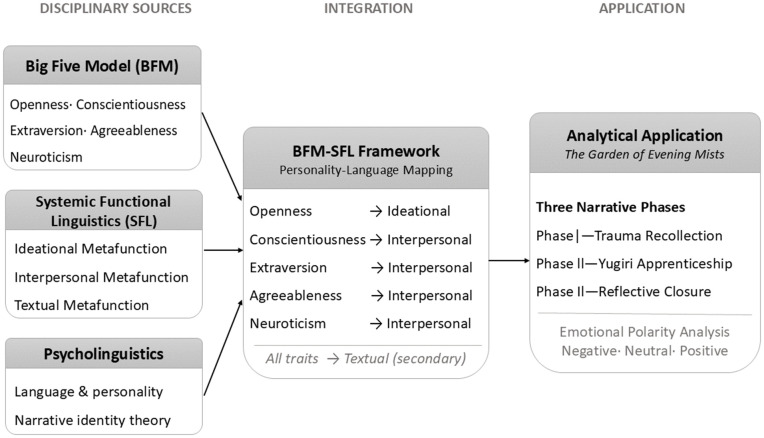
Construction of the Theoretical Framework Three disciplinary sources: the Big Five Model of Personality (BFM) [8,15], Systemic Functional Linguistics (SFL) [5] and psycholinguistic theory [7,14] converge into an integrated BFM–SFL mapping framework connecting each personality dimension to its primary metafunctional site of linguistic realization. This framework is applied to the qualitative discourse analysis of *The Garden of Evening Mists* across three narrative phases, with emotional polarity (negative, neutral, positive) as the primary analytical lens.

Together, these frameworks suggest that personality is both structured and narratively dynamic. The integration of psychological and linguistic theory enables personality traits to be operationalized as discernible linguistic patterns, providing a bridge between qualitative character interpretation and systematic discourse analysis. This theoretical link underpins the analytical framework presented in later sections and is visualized in Fig 1, which outlines the conceptual framework of this study.

#### 2.1.2. Systemic Functional Linguistics and Emotional Representation.

Systemic Functional Linguistics (SFL) provides the linguistic framework through which emotion and personality are observed in textual form. It conceptualizes language as a meaning-making system organized through three metafunctions, namely ideational, interpersonal, and textual which respectively represent experience, attitude, and discourse organization [5,22]. SFL conceptualizes language as a meaning-making system organized through three metafunctions that operate simultaneously in every clause, providing a comprehensive analytical resource for the study of language in social and psychological contexts [5,23]. Recent scholarship has confirmed the wide applicability and flexibility of SFL as an analytical tool across genres and languages, with particular attention to the interpersonal metafunction as a site of evaluative and affective meaning [24].

SFL’s focus on the interpersonal metafunction is particularly relevant to emotional expression. Mood, modality, and appraisal reveal how speakers or narrators encode evaluation and stance, forming systematic patterns that reflect emotional polarity and psychological disposition [6,25]. In *The Garden of Evening Mists*, these linguistic resources, including modal verbs, affective adjectives, and cohesive conjunctions, trace the protagonist’s psychological transition from repression to reconciliation.

**Fig 2:**
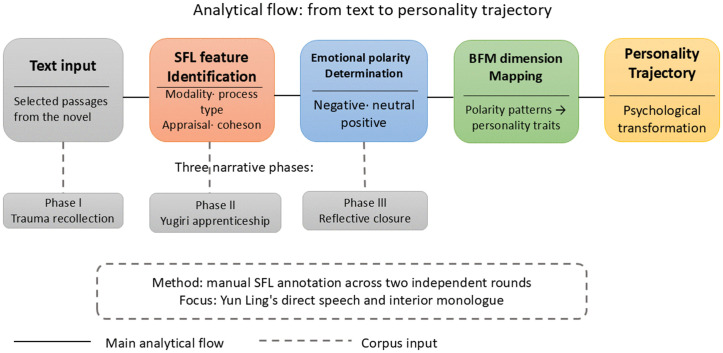
Analytical flow of the study Narrative passages from *The Garden of Evening Mists* are organized across three phases corresponding to the protagonist’s psychological trajectory. Each passage is annotated for SFL features at the ideational, interpersonal, and textual levels through manual analysis conducted across successive close readings. Emotional polarity (negative, neutral, positive) is determined on the basis of dominant lexicon grammatical patterns. Polarity orientations are then mapped onto Big Five personality dimensions to trace the protagonist’s personality trajectory across the narrative arc.

By linking SFL’s metafunctional categories to the BFM’s personality dimensions, this study constructs an interdisciplinary mapping that connects psychological structure with linguistic realization. Fig 2 visualizes how each of the five personality traits corresponds to specific metafunctional components, establishing a linguistic foundation for SFL-based close reading in subsequent analysis.

SFL thus serves as both a descriptive and operational framework, bridging linguistic form and psychological meaning. Through its integration with personality theory, it enables the present research to examine how emotion and personality are encoded in language, a necessary foundation for the qualitative discourse analysis that follows.

### 2.2. Analytical Method: SFL-Based Qualitative Discourse Analysis

The present study employs a qualitative discourse analysis approach grounded in Systemic Functional Linguistics (SFL) to examine how emotional polarity and personality are realized in the language of *The Garden of Evening Mists*. Rather than treating emotion as a surface lexical property, this framework attends to the grammatical and discoursal resources through which affective meaning is systematically constructed across the narrative.

A methodological caveat is in order before the analytical apparatus is described in detail. The Big Five framework was developed and validated on data from real persons producing real discourse, and SFL likewise theorizes language as the situated practice of speaking subjects. Their joint application to a fictional first-person narrator therefore involves an ontological transposition: linguistic patterns produced by the implied author of a literary work are read as indices of a personality structure attributed to a fictional consciousness. This transposition is not neutral, and recent work has begun to address it explicitly. Du and his colleagues argue that personality concepts can be meaningfully traced in literary discourse provided the analyst treats narrative voice as a structured discursive position rather than a transparent window onto interior states [2]; Bauditz and his colleagues similarly demonstrate that BFM-coherent linguistic patterns are detectable in literary fiction while cautioning that such patterns characterize the textual instantiation of personhood, not the psychological state of any actual individual [3]. The present study adopts this position. The personality trajectory traced through Yun Ling’s discourse is treated as a property of how the narrative constructs psychological coherence at the level of language, rather than as an inference about a real-world psyche. Within this frame, BFM-SFL analysis becomes a method for reading the linguistic architecture of fictional selfhood, with the limits of that reading set by the textual evidence the narrative makes available.

Emotional polarity is operationalized as the dominant affective orientation of a given passage, positive, neutral, or negative, determined by the convergence of three sets of SFL-derived linguistic indicators. Negative polarity is characterized by obligation modals (must, cannot, will not), negation structures, syntactic compression, and affective lexis of withdrawal or restraint. Neutral polarity is marked by evaluative nouns that express aesthetic or cognitive judgment, balanced clause structures that link perception and reflection, and descriptive cohesion without affective intensification. Positive polarity is realized through relational and material process verbs (restore, touch, plant), permissive modality (may, let, will), and evaluative lexis of acceptance and reconciliation. These criteria were applied consistently across the corpus.

Building on this framework, the present analysis proceeds through three metafunctional levels. At the interpersonal level, mood type, modality, appraisal values, and graduation are examined to identify how the narrator encodes emotional stance and psychological disposition toward events and interlocutors. At the ideational level, process types, namely mental, behavioral, material, and relational, are analyzed to trace shifts in the protagonist’s relation to memory, agency, and embodied experience. At the textual level, cohesive devices, thematic structure, and clause-complex patterns are examined to reveal how emotional continuity and disruption are organized across the discourse.

The mapping between BFM dimensions and SFL metafunctions presented in Table 1 is not a stipulated correspondence but a theoretical inference grounded in two convergent premises. The first is that personality, in its empirically validated formulations, is constituted by psych behavioural tendencies that find their fullest expression in observable language behaviour [2,3,14,15]. The second is that SFL articulates language behaviour into three simultaneous metafunctions, ideational (representing experience), interpersonal (negotiating relations), and textual (organizing discourse), each realized through distinct lexicogrammatical resources [5,23]. Bringing these premises together yields a principled expectation: a personality dimension defined primarily by a particular kind of psych behavioural tendency will be most densely realized in the metafunctional site whose grammatical resources most directly enact that kind of tendency.

Following this logic, Conscientiousness, defined in the BFM as the disposition toward self-regulation, restraint, and goal-directed control, is most densely realized at the interpersonal level, since self-regulation is grammatically enacted through deontic modality (must, cannot, will not), negation, and the constraint of one’s own propositional commitments. Process types in the ideational metafunction record what one does, but it is interpersonal modality that records what one holds oneself accountable to do or not do; this is the grammatical site at which Conscientiousness leaves its most diagnostic trace. Neuroticism, defined by emotional sensitivity and affective vulnerability, is similarly concentrated at the interpersonal level, but in a different grammatical region: not deontic modality but the appraisal system, particularly Affect [25] and graduation resources that intensify or mitigate emotional valence. Openness, defined by perceptual sensitivity, aesthetic engagement, and cognitive flexibility, has its principal site at the ideational level through process-type diversity (especially mental and behavioural processes of perception) and aesthetic evaluative lexis, with secondary realization at the interpersonal level through permissive modality (may, can, let) that grammatically enacts openness to possibility.

Agreeableness and Extraversion, as the two most thoroughly social dimensions of the BFM, are realized predominantly at the interpersonal level. Agreeableness is encoded through politeness strategies [19], cooperative material processes shared with co-participants, and the appraisal subsystem of Judgement; its trajectory in any given text is traceable through the patterning of these resources across stretches of dyadic interaction. Extraversion is realized through mood alternation, exclamative force, the pace and density of turn-taking, and the lexicogrammar of social engagement more broadly. Their density of realization in any particular text depends on whether the genre and narrative form structurally activate the interpersonal sites at which these dimensions live: a first-person introspective novel will activate Agreeableness through its sustained dyadic exchanges (with their built-in politeness, cooperation, and appraisal patterns) more fully than it activates Extraversion (which depends on the conversational pace and exclamative range that introspective narration tends to constrain). The mapping framework thus predicts not uniform density across the five dimensions but a textually conditioned pattern of activation, with the dominant grammatical site of each dimension serving as the principal locus of analytical attention.

The metafunction labels in Table 1, accordingly, designate the principal site of grammatical realization for each dimension, not its only site. The metafunctions operate simultaneously in every clause [5,21]; any personality dimension will leave traces in all three. The mapping does not partition language into mutually exclusive zones but identifies, for each BFM dimension, the metafunctional site at which its psychobehavioural definition is most directly enacted in lexicogrammar, and therefore the site at which qualitative analysis can most diagnostically read personality from text.

The mapping is therefore offered as an exploratory analytical framework rather than a validated model. Its warrant in the present study is demonstrative (it shows what such an analysis can yield when applied to a single literary text), and no claim is made for its general applicability across genres, authors, or traditions; that would require the broader, multi-text investigation noted in the Conclusion.

A key methodological consideration concerns the distinction between narrative voices in this first-person retrospective novel. The analysis focuses primarily on Yun Ling’s direct speech and interior monologue, in which the narrator and protagonist voices converge most directly. Passages of purely descriptive narration and dialogue involving secondary characters are treated as contextual evidence rather than primary analytical data, and the boundary between narrator and character perspective is explicitly noted in the close readings presented in 3.3.

The corpus was organized into three narrative phases corresponding to the protagonist’s psychological trajectory, namely trauma recollection, apprenticeship in Yugiri, and reflective closure, and passages were selected to represent a balance of narrative monologue, dialogue, and descriptive mediation within each phase. The author analysed each selected passage in successive close readings to check the consistency of the interpretive categories. The limitations of single-analyst annotation are acknowledged: the readings offered in this study are theoretically informed interpretations rather than independently validated classifications, and the analytical claims they support are correspondingly modal rather than categorical. This methodological position is consonant with the broader tradition of close reading in literary studies, in which the analyst’s transparency about framework, evidence, and inference takes the place of inter-rater agreement as the warrant for interpretive validity.

## 3. Results and analysis

### 3.1. Corpus selection and data preprocessing

The corpus for this study consists of selected narrative and dialogic passages from *The Garden of Evening Mists* [26], a novel characterized by its first-person retrospection and its interweaving of memory, trauma, and aesthetic contemplation. The text’s highly reflective narration and balanced use of silence, metaphor, and sensory imagery make it particularly suitable for emotion-oriented and personality-aware linguistic analysis. The passages selected for close reading and discourse analysis emphasize moments of emotional intensity and psychological transition, those in which Yun Ling’s introspection intersects with her interactions with Aritomo and the remembered past. Such selections ensure that both narrative voice and character psychology remain stable analytical anchors across the corpus.

Passage selection followed a purposive rather than exhaustive logic, consistent with the close-reading tradition in literary stylistics. Rather than sampling the novel at fixed intervals or aiming at quantitative coverage, the analysis identified passages on the basis of two interpretive criteria: emotional intensity (moments where affective meaning is most densely realized in the lexicogrammar) and psychological transition (moments that mark or enact shifts in Yun Ling’s relation to memory, others, and the remembered past). Within each of the three narrative phases, passages meeting these criteria were drawn from across the relevant stretch of the novel so as to represent narrative monologue, dialogue, and descriptive mediation rather than any single discourse mode. Because the analysis is qualitative and demonstrative, the passages discussed in Sections 3.3 and 3.4 are presented as representative instances of each phase’s dominant orientation rather than as an exhaustive inventory; the warrant for the reading rests on the transparency of the selection criteria and the analytical detail brought to each passage, not on corpus size.

To achieve interpretive coherence, the corpus was organized across three narrative phases, trauma recollection, apprenticeship in Yugiri, and reflective closure, mirroring the psychological trajectory of the protagonist. Within each phase, excerpts were sampled to preserve a proportional representation of narrative monologue, dialogue, and descriptive mediation. This stratified sampling aligns with the methodological principle that emotion in literary discourse is contextually embedded rather than lexically isolated [11,22]. To support this framework, the corpus is divided into major phases corresponding to the protagonist’s psychological trajectory. Each phase reflects a distinct emotional and linguistic configuration within the novel. Table 2 presents the segmentation of the narrative corpus from *The Garden of Evening Mists* by psychological phase and linguistic focus.

**Table 2:** Segmentation of the Narrative Corpus from *The Garden of Evening Mists* by Psychological Phase and Linguistic Focus Phases correspond to the protagonist Yun Ling’s psychological trajectory across *The Garden of Evening Mists*. Passages were selected to preserve a proportional representation of narrative monologue, dialogue, and descriptive mediation within each phase, consistent with the principle that emotion in literary discourse is contextually embedded rather than lexically isolated [11,23]. The corpus focuses on Yun Ling’s direct speech and interior monologue, where the narrator’s and character’s voices converge most directly.

Narrative Phase	Segment Type	Key Emotional Focus	Sampling Notes
**Trauma Recollection**	Interior monologue, flashback narration	Guilt, repression, survivor’s silence	Dense affective language; self-narration dominates; obligation modals and negation structures prevalent
**Yugiri Apprenticeship**	Dialogues, reflective description	Discipline, serenity, restrained affection	Frequent interpersonal modality; presence of Aritomo as interlocutor; evaluative lexis of proportion and harmony
**Reflective Closure**	Retrospective reflection, temporal shifts	Acceptance, reconciliation, mortality awareness	Elevated evaluative lexis and metaphorical realization; material and relational process verbs; permissive modality

The analytical procedure preserves the expressive variety of the novel intact, so that differences in style across the three narrative phases remain visible and analytically meaningful. This balance makes it possible to engage with the text consistently without losing its literary texture. Each selected passage can therefore be read as a small linguistic window into Yun Ling’s changing state of mind; tracing how emotional polarity shifts through the course of her story. In this respect, the selection and organization of the corpus is not merely a preparatory step but is itself part of the interpretive process, ensuring that the analytical material remains faithful to the novel’s tone and meaning while providing a structured basis for the SFL-based close reading that follows.

### 3.2. Dominant emotional polarity across narrative phases

**Fig 3:**
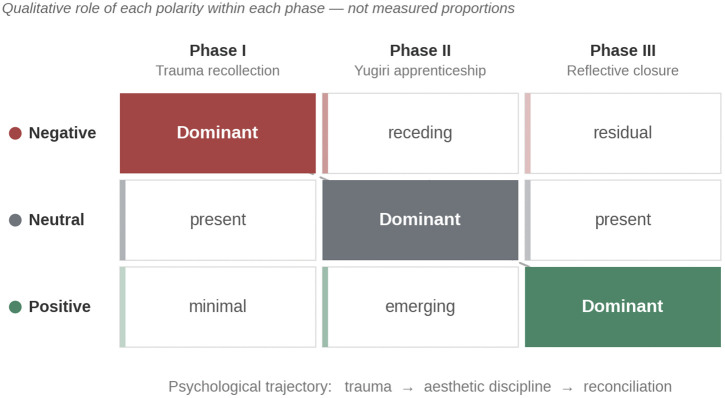
Dominant Emotional Polarity across Narrative Phases in *The Garden of Evening Mists* Dominant emotional polarity across the three narrative phases of *The Garden of Evening Mists.* Each cell describes the qualitative role of a polarity category within a given phase, as identified through SFL-based close reading; the matrix represents interpretive orientation, not measured proportions, and no cell encodes a quantitative value. Negative polarity is dominant in Phase I (Trauma Recollection), where it is realized through obligation modals, negation structures, and paratactic fragmentation; neutral polarity is dominant in Phase II (Yugiri Apprenticeship), marked by evaluative balance, aesthetic reflection, and stabilized interpersonal modality; positive polarity is dominant in Phase III (Reflective Closure), realized through relational processes, permissive modality, and declarative acceptance. The diagonal indicates the overall direction of psychological transformation across the narrative arc (trauma → aesthetic discipline → reconciliation).

The analysis of selected passages from *The Garden of Evening Mists* reveals a discernible pattern of emotional polarity across the three narrative phases, reflecting the protagonist’s psychological trajectory from trauma and repression toward reconciliation and acceptance. Rather than assigning polarity through predetermined categorical labels, it describes the dominant affective orientation of each phase as identified through SFL-based close reading, establishing the interpretive foundation for the detailed linguistic analysis. The dominant affective orientation of each phase is summarized in Fig 3.

In the trauma recollection phase, negative polarity predominates. The narrative voice is characterized by self-imposed restraint, foreclosed possibility, and the persistent intrusion of unassimilated memory. Yun Ling’s language consistently resists elaboration and reconciliation, returning instead to what cannot be said, what cannot be healed, and what cannot be recovered. Linguistic markers of negative polarity, such as obligation modals, negation structures, paratactic fragmentation, and affective lexis of withdrawal, cluster densely in passages of introspection and flashback, producing a textual environment in which silence functions not as absence but as the dominant communicative mode.

The Yugiri apprenticeship phase is marked by a shift toward neutral polarity. As Yun Ling engages in the disciplined practice of garden-making under Aritomo’s instruction, the narrative language moves away from the recursive self-examination of the trauma phase toward evaluative description and aesthetic reflection. Emotional intensity is neither suppressed nor freely expressed but channeled into the vocabulary of proportion, composition, and craft. The interpersonal register softens, obligation modals give way to epistemic modality, and the thematic progression of the prose becomes more linear and purposeful. This neutral phase is not emotionally empty; rather, it represents a form of psychological steadiness in which feeling is held in disciplined equilibrium.

In the reflective closure phase, positive polarity becomes increasingly dominant as the narrative language turns outward through material and relational processes. Yun Ling’s voice acquires a declarative quality that was absent from the earlier phases: she no longer interrogates or forecloses but asserts, accepts, and integrates. Modal verbs of permission and possibility replace the earlier negations, and the evaluative lexis shifts from absence and loss toward release and restoration. The emotional movement of this phase is not one of triumphant resolution but of quiet acceptance, a psychological reconciliation realized through the formal resources of language rather than through any dramatic narrative event.

These three phases trace a coherent emotional arc: from the negative polarity of trauma-related self-regulation, through the neutral polarity of disciplined reappraisal, to the positive polarity of reconciliation and acceptance. This progression corresponds to the personality trajectory described in the BFM-SFL framework: the gradual modulation of high Conscientiousness and high Neuroticism toward increased Openness and reduced neurotic tension. The following section examines the specific linguistic mechanisms through which this trajectory is realized at the interpersonal, ideational, and textual levels of the narrative.

### 3.3. Linguistic interpretation of emotional polarity

The emotional polarity patterns identified across the three narrative phases are interpreted through the lens of Systemic Functional Linguistics (SFL), which explains how language realizes emotion and personality through ideational, interpersonal, and textual choices. This section connects linguistic evidence drawn directly from *The Garden of Evening Mists* to affective meaning, demonstrating how each metafunctional level contributes to the construction of emotional polarity and its corresponding personality dimensions. Table 3 summarizes representative examples illustrating how emotional stance is encoded in clause structure, process type, and cohesive rhythm across the three narrative phases.

**Table 3:** Dominant Linguistic Features by Emotional Polarity in *The Garden of Evening Mists* Representative examples are drawn from *The Garden of Evening Mists* [26]. SFL metafunctions indicate the primary analytical level through which each polarity category is realized; metafunctions operate simultaneously in every clause and the designations shown represent dominant rather than exclusive associations [5]. Emotional polarity criteria follow the operationalization described in Section 2.2.

Emotional Polarity	Dominant Linguistic Features	SFL Metafunction	Representative Example
**Negative**	Affective lexis of pain and withdrawal (*painful silence*, *grief*, *horror*) Obligation modals (*must*, *cannot*, *never*) Paratactic clause structure; negation structures; interpersonal markers of detachment	Interpersonal / Textual	“He looked at me, then turned and walked away, wedging a *painful silence* into the space between us.” (p. 93) “What words could have healed my pain, returned my sister to me? None.” (p. 13)
**Neutral**	Evaluative nouns (*silence*, *stillness*, *proportion*, *composition*) Descriptive phrasing; balanced clause structures linking perception and thought; reflective modality	Interpersonal / Ideational	“Even the silence of the road would halt me. But how does one capture stillness on paper?” (p. 145) “We are composing a picture within this frame … they have a sense of uncertainty, of tension and possibility.” (p. 95)
**Positive**	Relational and material process verbs (*restore*, *release*, *let go*, *plant*) Permissive modality (*may*, *can*, *will*) Declarative mood; ideational coherence through process-type continuity; evaluative lexis of acceptance and reconciliation	Interpersonal/Ideational	“I let it go, and I feel I am releasing a bird from my grasp.” (p. 326) “My decision to restore the garden is the correct one, the only one I can make.” (p. 327)

The metafunction labels in Table 3 indicate the principal sites at which each emotional polarity is grammatically realized, not exclusive locations. Because the metafunctions operate simultaneously in every clause [5,23] any given polarity will leave traces across all three; the labels identify where the densest and most diagnostic linguistic evidence is found. Negative polarity is concentrated at the interpersonal level through obligation modals and negation, with textual support from paratactic compression that fragments cohesive flow. Neutral polarity foregrounds interpersonal evaluation through reflective modality and ideational representation through aesthetic process types and evaluative nouns. Positive polarity is realized most distinctively through interpersonal resources, permissive modality and declarative mood, which encode openness to possibility and acceptance of contingency, supported by ideational continuity in relational and material process types that enact reconciliation as embodied agency. The shift from negative to positive polarity is thus not merely a change in lexical valence but a systematic redistribution of meaning across the metafunctions, with the interpersonal site of modality serving as the principal grammatical theatre in which Yun Ling’s psychological transformation is staged.

#### 3.3.1. Interpersonal function: Modality and appraisal.

The interpersonal metafunction provides the clearest linguistic window into Yun Ling’s emotional stance across the novel, revealing how modality and appraisal resources encode psychological disposition at the level of grammar rather than explicit declaration. Recent scholarship has extended the application of Appraisal theory to literary writing contexts, demonstrating that skilled interpreters of literary texts deploy Appreciation, Engagement, and Graduation resources to realize nuanced evaluative stances [16]. In the trauma recollection phase, the interpersonal register is characterized by declarative clauses that foreclose possibility rather than open it. The novel’s opening establishes this pattern immediately: “What words could have healed my pain, returned my sister to me? None” (p. 13). The rhetorical question followed by the monosyllabic negative answer enacts through syntax the very closure it describes. There is no modal hedging here, no epistemic uncertainty: only the flat assertion of impossibility. The absence of obligation modals is itself significant: Yun Ling does not say she cannot be healed; she says there are no words that could heal her. This shift from deontic to conditional modality positions the impossibility as inherent in the world rather than self-imposed, yet the effect is the same, an emotional foreclosure that aligns with high Conscientiousness and high Neuroticism, traits associated with moral rigidity and the suppression of affect [13].

Appraisal resources in these passages operate through negative affect inscribed in first-person constructions. When Yun Ling acknowledges that “the smell... I thought I had forgotten the smell. But one never does” (p. 83), the generalization encoded in the impersonal pronoun one universalizes her individual trauma into a categorical truth, simultaneously distancing the self from the experience and asserting its permanence. The modal never closes off any possibility of escape, and the shift from first-person to impersonal subject performs a dissociative movement characteristic of trauma discourse: the self becomes a case of a general rule rather than a feeling individual. The relationship between trauma and language is not merely one of content but of form: trauma shapes the very capacity and inclination to speak, generating patterns of withholding, fragmentation, and silence that are as linguistically meaningful as overt affective expression [27].

As the narrative moves into the Yugiri apprenticeship phase, the interpersonal register shifts toward evaluative language centered on aesthetic judgment and proportion. Aritomo’s instruction: “We are composing a picture within this frame... they have a sense of uncertainty, of tension and possibility.” (p. 94), introduces a collaborative first-person plural that draws Yun Ling into a shared aesthetic enterprise. The inclusive we replace the isolated I of the trauma passages, and the evaluative nouns uncertainty, tension, and possibility reframe psychological instability as aesthetic virtue. Crucially, possibility appears here for the first time as a positive value, directly inverting the foreclosed possibilities of the trauma phase. This shift in appraisal, from negative affect directed inward to positive appreciation directed outward, signals the emergence of Openness alongside the stabilization of Conscientiousness.

In the reflective closure phase, positive polarity is realized through declarative constructions that carry the weight of hard-won acceptance. The statement “Nature is the best teacher” (p. 305), reported as Aritomo’s words to Yun Ling, is syntactically unguarded: no modal qualification, no negation, no hedging of any kind. The relational process asserts identity with finality. More significantly, Yun Ling does not resist or qualify this statement in her narration: she allows it to stand as wisdom. This receptivity itself marks a shift in her interpersonal stance, from a voice that questions and forecloses to one that receives and integrates. The culminating declaration: “I know now that whether it was an accident or if he did it on purpose, there was nothing I could have said or done to have prevented it” (p. 325), completes this arc. The epistemic modal know replaces the earlier interrogatives and negations, and the conditional clause that follows it acknowledges contingency without being destabilized by it. This is the interpersonal grammar of acceptance: not the absence of doubt, but its containment within a larger declarative frame.

#### 3.3.2. Ideational function: Process types and experiential meaning.

At the ideational level, emotional polarity is realized through contrasting process types that trace Yun Ling’s shifting relation to memory, agency, and embodied experience. The progression from mental and behavioral processes in the trauma phase to material and relational processes in the closure phase enacts the novel’s central psychological movement at the level of grammar itself.

In the trauma recollection phase, mental processes dominate the narrative voice. The act of memory is rendered as a form of involuntary perception: Yun Ling does not choose to remember but finds herself remembering, and the linguistic form of this experience reflects its compulsory quality. When she describes emerging from the internment camp: “I was frail when I emerged from the slave-labor camp, and my health has never recovered completely.” (p. 34), the stative verb was, and the present perfect has never recovered together suspend the past in the present, making the damage continuous and ongoing rather than completed. The experiential meaning here is that trauma does not end; it persists as a condition of the self. The mental process of recollection is not chosen but imposed, as when the sight of origami flowers causes an involuntary memory of the camp: “I pushed the memory away” (p. 42). The behavioral process pushed away is striking precisely because it applies a physical action to a mental experience: memory is treated as a material object that can be displaced, yet the effort required to do so reveals how resistant it is to displacement.

Behavioral processes appear alongside mental ones in the early chapters to create a portrait of a self that monitors its own responses. Yun Ling does not simply feel: she watches herself feeling, intervening in her own emotional process as a form of conscientious self-regulation. This reflexive quality, in which the experiencing self and the observed self converge, is characteristic of the high-Conscientiousness, high-Neuroticism personality constellation: emotion is not expressed but managed, and the management is itself a form of expression [28]. The transition to material processes in the apprenticeship phase marks a decisive shift in the protagonist’s experiential orientation. Physical labor becomes the medium through which psychological reappraisal is affected: Yun Ling digs, rakes, plants, and lifts stones under Aritomo’s instruction, and these material processes externalize the internal work of recovery. The soil is not merely soil: it is the material through which grief is metabolized into action. When Aritomo tells her, “The girl who had once walked in the gardens of Kyoto with her sister... she is still there. Deep inside, she is still there” (p. 86), the spatial metaphor deep inside positions selfhood as something buried rather than destroyed, recoverable through the process of excavation. The garden, as both a literal site and a psychological metaphor, becomes the space in which material and mental processes converge. The capacity to construct new meaning from repeated engagement with difficult experiences, transforming the act of recollection into the act of re-making, is central to narrative identity development [29].

In the reflective closure phase, relational processes emerge with increasing frequency, encoding a self that defines itself through connection rather than isolation. The novel’s final movement centers on an act of deliberate release: “I let it go, and I feel I am releasing a bird from my grasp” (p. 326). The material process let go is accompanied by a mental process of perception (I feel) that grounds the physical act in subjective experience, binding action and awareness in a single clause. The metaphor of the bird transforms an act of relinquishment into one of liberation: not loss but release, not emptiness but openness. This is the ideational grammar of reconciliation: the self that once pushed memory away now lets it go, and the difference between these two processes, one defensive and one voluntary, encodes the full arc of Yun Ling’s psychological transformation.

#### 3.3.3. Textual function: Thematic structure and cohesion.

At the textual level, emotional polarity is expressed through shifts in cohesive organization and thematic progression that mirror the protagonist’s psychological state. The formal properties of Tan’s novel, its syntactic rhythms, its patterns of repetition and variation, its management of silence and speech, encode emotional meaning at the level of discourse organization. In passages of negative polarity, the dominant textual pattern is negative cohesion: the text coheres around what is missing rather than what is present, and the resulting voice defines itself by what it has lost. The early courtroom sequence, in which Yun Ling listens to her own life being recounted at her retirement, offers a paradigmatic instance:

“I had never spoken of the three years I had spent in the camp to anyone. I tried not to think about it as I went about my days, and mostly I succeeded. But occasionally the memories still found their way in, through a sound I heard, a word someone uttered, or a smell I caught in the street.” (p. 17)

Three textual mechanisms work together here. First, the cohesive chain is built almost entirely from negative and limiting elements: *never, not, mostly* (a hedged success), *but occasionally* (a concessive reversal). The lexical chain that holds the passage together is one of foreclosure and qualified failure rather than affirmation. Second, the referent of the trauma is never named directly: it appears only as anaphoric *it* (“not to think about *it*”, “memories still found their way in”). The text’s textual reference grammar enacts what its propositional content describes: the experience is grammatically present but lexically withheld, holding its place in the discourse without entering it. Third, the thematic progression is deflected rather than developmental: each clause introduces an attempt at containment (*I had never spoken... I tried not to think... mostly I succeeded*) and the final clause undoes the containment without resolving it (*But occasionally the memories still found their way in*). The textual arc is not a movement from problem to resolution but a recursive holding pattern: the same effort repeated, the same partial failure repeated. This is the textual grammar of trauma: cohesion through what is refused, themed through what cannot be progressed past.

The same negative cohesive logic operates across the trauma passages of Phase I more broadly. The semantic fields of silence, forgetting, and incompleteness recur in clustered repetition: *never, no, none, not, pushed away,* producing a discourse that is held together by absence rather than presence. Sentence fragments and paratactic structures reinforce the same effect at the level of clause organization, refusing the syntactic subordination that would integrate experience into a continuous narrative. What emerges, textually, is a voice that has not yet learned to organize its own past.

The apprenticeship phase introduces a markedly different textual organization. Aritomo’s speech is characterized by complex clause structures that embed one thought within another, modeling a form of cognitive integration that Yun Ling gradually internalizes. His instruction: “We are composing a picture within this frame... they have large tracts of emptiness, their composition is asymmetrical... they have a sense of uncertainty, of tension and possibility.” (p. 94), moves through assertion, elaboration, and reframing in a single extended utterance. The ellipsis marks pause that are contemplative rather than evasive, and the final evaluative nouns, uncertainty, tension, possibility, introduce positive value into what might otherwise be experienced as deficiency. Yun Ling’s own narration begins to adopt these structural habits: longer sentences, more frequent subordination, a thematic progression that moves toward rather than away from meaning.

The reflective closure phase is marked by the most complex and integrated textual organization in the novel. The penultimate scene, in which Yun Ling releases a lantern over the pond, moves through a series of precisely coordinated clauses: “I light the candle in the lantern and hold it in my hands. I close my eyes and see Aritomo. A woman’s face appears beneath my eyelids, and I realize it is Yun Hong. She does not smile. She is not angry; she is not sad. She is only a memory” (p. 325). The clauses are short but syntactically complete, each one resolved before the next begins, a thematic progression that is forward-moving and purposeful rather than circular and obsessive. The negations here, does not smile, is not angry, is not sad, no longer foreclose but classify, and the final clause she is only a memory achieves through the word only what the entire novel has been working toward: the containment of grief within a liveable category. Memory, finally, is only memory, still present, but no longer overwhelming.

The culminating declaration: “My decision to restore the garden is the correct one, the only one I can make.” (p. 326), closes the novel’s textual arc with a declarative sentence of complete syntactic and thematic integration. The modal can replace the earlier cannot and never; the evaluative adjective correct replaces the earlier interrogatives and negations; and the phrase the only one I can make transforms necessity from constraint into acceptance. This is the textual grammar of reconciliation: not the absence of limitation, but its transformation into choice.

### 3.4. Personality mapping and psychological analysis

The final stage of analysis correlates the dominant emotional polarity orientations identified in Section 3.2 with personality dimensions from the Big Five Model (BFM) of Personality. This mapping reveals how the protagonist’s emotional language and behavioral tendencies reflect underlying psychological traits and their transformation across the narrative. By integrating linguistic evidence with theoretical models of personality, the study situates literary character analysis within a theoretically informed interpretive framework, bridging stylistic evidence with psychological coherence.

#### 3.4.1. Personality dimensions reflected in emotional polarity.

Yun Ling’s linguistic behavior throughout *The Garden of Evening Mists* can be read as tracing a gradual transformation from emotional suppression to reconciliation. In the trauma-recollection phase, negative polarity dominates. The language abounds with modal expressions of restraint, can’t, will not, never, as in her refusal to reenter a hospital:

“I’ll never put myself inside another one again. Never.” (p. 121)

and in her self-defensive response to companionship:

“You can’t live alone,” Frederik says. … “It’s too late for me to change my ways.” (p. 121)

Such constructions convey self-imposed duty and moral tension, aligning with a high-conscientiousness/high-neuroticism personality constellation often linked to survivor guilt [12,13]. The repetition and negation pattern rhythmically enact Yun Ling’s internal struggle to maintain order amid trauma.

During the Yugiri apprenticeship phase, emotional neutrality emerges. The language shifts toward evaluative lexis reflecting aesthetic judgment and proportion. Aritomo’s lesson,

“We are composing a picture within this frame. … Look at our paintings — they have large tracts of emptiness, their composition is asymmetrical … they have a sense of uncertainty, of tension and possibility.” (p. 95–96)

foregrounds balance and restraint, linguistic realizations of deliberation and cognitive control. Yun Ling’s reflective narration and descriptive precision (the soil felt cool and moist on my skin, p. 96) signal stabilized conscientiousness and growing openness. The apprenticeship’s aesthetic discipline thus becomes a metaphorical and linguistic medium for psychological reappraisal, echoing McAdams’s theory of coherence through narrative reconstruction [30].

In the reflective closure phase, positive polarity predominates as the narrative language turns outward through material and relational processes. The verbs dig, plant, restore, and touch reveal embodied reconciliation:

“I light three more joss sticks and plant them into the moist patch of soil in front of it, then watch the smoke rise into the trees.” (p. 321)

“It will be restored to the way it used to be, the way I remember it.” (p. 324)

Modal verbs of permission (may, let, will) replace negation, articulating release and acceptance. This linguistic and psychological convergence corresponds to high openness and reduced neuroticism, consistent with findings that emotional flexibility and coherence predict well-being in narrative identity. In this closing phase, Yun Ling’s language no longer guards against memory; it transforms it into understanding, completing the novel’s emotional arc of moral restraint toward inner peace.

#### 3.4.2. Personality trajectory across narrative phases.

The three narrative phases of *The Garden of Evening Mists* do not simply mark temporal divisions in the plot; they encode distinct configurations of personality expression, each characterized by a different balance of Conscientiousness, Neuroticism, and Openness. The trajectories of these dimensions across the narrative are traced here through close reading of the linguistic markers that index each trait within the SFL-based analytical framework. Conscientiousness is realized through obligation modals and negation structures encoding moral restraint (*must, cannot, will not, never, had to*); Neuroticism through the semantic field of emotional vulnerability and trauma (*pain, fear, dread, guilt, grief, afraid*); and Openness through markers of perceptual sensitivity, aesthetic appreciation, and psychological acceptance (*beautiful, wonder, peace, release, accept, restore, nature*). These linguistic categories are interpretive rather than enumerative: their function is to direct close reading toward the formal sites where personality is grammatically realized, not to support quantitative claims.

Of the three dimensions, Conscientiousness is the most stable and consistently prominent throughout the narrative. Its sustained, gradually intensifying presence across all three phases suggests that disciplined self-regulation reads less as a reactive response to trauma than as a foundational feature of how the narrative constructs Yun Ling’s psychological identity, present from the opening pages and persisting through to the novel’s close. What shifts is not the presence of this trait but its affective valence. In Phase I, the obligation modals and negation structures that index Conscientiousness encode suppression and self-imposed restraint: *cannot*, *will not*, *never* are the grammatical instruments of a self that has armoured itself against feeling. By Phase III, the same disciplined register has been transformed. The declaration “My decision to restore the garden is the correct one, the only one I can make” (p. 327) retains the conscientious insistence on moral clarity, but the modal *can* has replaced *cannot*, and necessity has been reframed as agency. Conscientiousness, in the novel’s closing movement, is no longer a form of self-protection but of self-determination.

Neuroticism traces the most nuanced arc of the three dimensions: a discernible recession through the Yugiri apprenticeship phase, followed by a partial return in the closing movement. Its decline through the apprenticeship phase reflects the disciplining of emotional vulnerability through aesthetic practice: as Yun Ling’s language shifts toward the evaluative lexis of proportion and harmony, the affective markers of fear and pain recede into the background of the prose. The minor recovery in Phase III is equally revealing and should not be misread as regression. It registers the emotional weight of genuine reconciliation: releasing the lantern, accepting Aritomo’s disappearance, confronting what cannot be recovered or explained. “The smell... I thought I had forgotten the smell. But one never does.” (p. 83): grief in the closing phase is not eliminated but integrated, its linguistic presence reduced yet legible, transformed from wound into witness. The slight uptick in Neuroticism markers is the formal trace of this integration, a psychologically honest acknowledgement that reconciliation carries its own affective cost.

Openness displays the most dramatic trajectory of the three dimensions, and its pattern is the most interpretively complex. Its sustained presence across Phases I and II, remaining textually conspicuous even within the constrained emotional register of the trauma phase, suggests that perceptual and aesthetic sensitivity is not a capacity that Yun Ling develops through the apprenticeship but one she already possesses and has never fully relinquished. She attends to light, silence, and the textures of the natural world even when she cannot receive them without pain; she notices beauty even as she refuses to be consoled by it. What the sharp rise in Phase III registers, then, is not the emergence of Openness but its liberation: the removal of the psychological constraints that had previously limited its expression. This trajectory is realized across multiple sites in Phase III: the deliberate movement to Yugiri’s restoration, the dialogues with Tatsuji, and the closing reflections on Aritomo. Its most concentrated textual culmination, however, is the lantern-release passage: “I let it go, and I feel I am releasing a bird from my grasp.” (p. 326). Here three grammatical movements coincide: the material process let go releases physical and psychological hold; the mental process I feel registers the subject’s awareness of that release as openness rather than loss; and the metonymic substitution of the lantern for Yun Hong’s memory enacts the long-deferred conscientious commitment to her sister. The clause is the moment at which all three personality dimensions, Openness, Neuroticism, Conscientiousness, are seen to be in motion together, no longer in tension.

#### 3.4.3. Interpersonal trajectory: Agreeableness and extraversion in Yun Ling’s Voice.

While the Conscientiousness–Neuroticism–Openness configuration captures the most densely realized personality dimensions in the novel, the BFM framework, as instantiated in Table 1, also encompasses Agreeableness and Extraversion. Both dimensions are present in Yun Ling’s discourse, though with markedly different densities and through different grammatical sites. Agreeableness traces the most consequential interpersonal arc in the novel, since the narrative’s central thematic question, whether and how to release inherited hatred, is enacted at the interpersonal level of language. Extraversion, by contrast, is realized at lower density and in a constrained register characteristic of introspective first-person narrative; nevertheless, its trajectory across the three phases is legible and reinforces the interpretive arc established by the other dimensions.

In Phase I, Yun Ling’s language is saturated with the markers of low Agreeableness: refusal, withdrawal, and the grammatical foreclosure of cooperative engagement. The lexical field of refused, hatred, cold, and anger, which together account for over fifty tokens across the novel and concentrate disproportionately in the early chapters, encodes not merely emotional state but a consistent orientation toward others as objects of judgement rather than subjects of trust. The interpersonal grammar that realizes this stance is twofold. First, Yun Ling deploys imperative and high-deontic declarative mood toward Japanese interlocutors with no mitigation: “I will make sure all copies of your book are pulped” (p.31) and “I will decide what the Japanese people have a right to” (p.32) combine the deontic will with first-person agency to enact what Brown and Levinson [19] term a maximal face-threatening act, performed without any of the redress that would index Agreeableness. Second, when others extend the cooperative move that would invite reciprocal Agreeableness, most notably Magnus’s appeal “This hatred in you, you can’t let it affect your life” (p. 51), Yun Ling’s response refuses uptake: “It is not up to me, Magnus” (p. 51). The grammatical pattern here is diagnostic: an existential construction that displaces agency outside the self, denying the very capacity for the cooperative repositioning Magnus proposes. Magnus’s subsequent extended self-disclosure of his own war-bereaved family, culminating in the more emphatic appeal, “Let it go, this hatred in you. Let it go” (p. 52), is met with no recorded reply at all. This silence completes the refusal of uptake at a deeper structural level than the earlier verbal demurral.

What renders the Agreeableness trajectory analytically rich is that the novel structurally positions Magnus as the persistent interlocutor through whom the disagreeable stance is interrogated. Magnus’s own self-disclosure: “I held on to my hatred for forty-six years” (p. 52), follows the model of overcoming resentment that Yun Ling resists for the bulk of the narrative. The interpersonal grammar of these exchanges, with their pattern of cooperative offer and refused uptake, makes Magnus the textual instrument by which Yun Ling’s low Agreeableness is rendered legible as a sustained psychological position rather than a mere lexical mood.

Phase II tracks a tentative repositioning enacted through cooperative material processes rather than through positive evaluative lexis. The apprenticeship at Yugiri proceeds through joint action verbs, working alongside Aritomo in the garden, transplanting, raking, observing, that constitute Agreeableness in its most literal SFL realization: cooperative material processes in which two participants share agency over a single goal. The novel makes few explicit declarations of warmth or trust during this phase (the lexis of trust, compassion, tenderness remains conspicuously sparse throughout), but this absence is itself diagnostic. Yun Ling’s Agreeableness in Phase II is realized not through the conventional appraisal lexis of warmth but through the experiential grammar of co-participation. This is the novel’s distinctive instantiation of the dimension: cooperation without sentiment, alliance without disclosure.

Phase III brings the Agreeableness arc to its most fully realized expression through the Yun Ling–Professor Yoshikawa Tatsuji relationship. The interpersonal trajectory here is exact: from the Phase III opening, where Yun Ling addresses Tatsuji with the same hostile deontic declaratives she directed at Japanese interlocutor’s decades earlier, through a sustained sequence of two weeks of conversation, to the closing exchange in which Tatsuji offers her the woodblock print and Yun Ling extends her hands to receive it: “Extending my hands, I receive the book from him. I feel we have known each other for longer than the two weeks he has spent here. We are the same.” (p.324). The grammatical movement is from imperative-mood threat through declarative-mood acknowledgement to the closing relational process clause *We are the same,* a high-Agreeableness identification with the interlocutor whom the entire narrative had previously constructed as an inadmissible other. That this identification is performed with a Japanese historian, in the closing pages, with a man whose presence at the war’s perimeter has just been disclosed, makes the Agreeableness arc inseparable from the novel’s central thematic resolution. Agreeableness is not an incidental dimension here; it is the linguistic site at which the narrative’s core ethical question is settled.

Extraversion presents a different analytical situation. The lexicogrammatical resources conventionally associated with high Extraversion, such as exclamative mood, rapid turn-taking, and assertive declaratives encoding social dominance, are sparse in Yun Ling’s discourse, as is consistent with first-person introspective narration shaped by trauma. This sparseness is not, however, equivalent to absence; rather, Extraversion in this text is realized through a constrained register whose trajectory across the three phases parallels the Agreeableness arc, though at lower density. In Phase I, Yun Ling’s interpersonal engagement is characterized by minimal-turn dialogue, abrupt declaratives, and a pattern of conversation closure rather than elaboration. In Phase II, the apprenticeship structure produces a different interpersonal pattern: declarative–interrogative alternation between Yun Ling and Aritomo, accompanied by extended joint silence as a marked communicative resource rather than a default state. In Phase III, the dialogues with Frederik in the present-time frame and with Tatsuji across his two-week stay are sustained, elaborated, and characterized by the cooperative turn-taking that signals the recovery of social engagement. The frequency with which Frederik appears in the opening and closing chapters, and the tonal ease of those exchanges relative to Phase I, register an Extraversion trajectory whose direction reinforces the broader interpretive claim: not the development of a previously absent capacity, but the gradual lifting of the constraints that had suppressed its expression.

That Agreeableness emerges as the most fully realized of the interpersonal dimensions, while Extraversion remains constrained, is not a defect of the text-personality fit but a structural feature of how introspective first-person narrative instantiates the BFM. The narrative form privileges the grammatical sites at which inner life negotiates its relation to others (modality, appraisal, politeness) over those at which sociality is performed in real time (mood alternation, exclamative force, conversational expansiveness). What the BFM framework registers, when applied to a text of this kind, is not all five dimensions equally but those which the genre most fully activates, a finding that supports rather than undermines the SFL-based mapping, since each dimension’s density of grammatical realization is itself diagnostic of the text’s interpersonal architecture.

#### 3.4.4. Interpretive implications.

The interaction between emotion and personality in *The Garden of Evening Mists* suggests that linguistic restraint operates not as emotional absence but as a strategy of psychological regulation. Yun Ling’s deliberate syntax and precise use of modal verbs enact a disciplined control over affect, turning language into a stabilizing mechanism when emotional coherence is at risk. Her gradual shift from modal necessity to modal possibility, and from fragmented parataxis to cohesive coordination, marks a subtle reconstruction of meaning. This linguistic evolution can be read as paralleling a psychodynamic process, moving from trauma containment toward emotional reintegration, consistent with empirical findings that lexical diversity and modal flexibility accompany recovery and cognitive openness [13,28].

The novel also exemplifies McAdams’s theory of narrative identity, which posits that selfhood is shaped through the act of narrating experience. Yun Ling’s linguistic self-discipline gradually transforms into reflective composure; her controlled expression becomes a medium of understanding rather than suppression. Through language, her personality development is not merely represented but realized: each grammatical choice contributes to the reconstruction of self and meaning. Empirical research confirms that narrative identity themes of agency and integration contribute independently to psychological well-being, over and above the effects of dispositional personality traits [31]. The novel’s closing movement enacts what McAdams describes as the shift from redemptive to acceptance narratives [32]. This movement toward acceptance is not a passive resignation but an active narrative achievement: a reorientation of selfhood that acknowledges irreversible loss without being defined by it [33]. Rather than transforming suffering into triumph, Yun Ling’s language moves toward a quieter accommodation of what cannot be changed, integrating loss into a liveable account of selfhood. The SFL-based analysis presented in this study demonstrates that close reading, when grounded in a systematic linguistic framework, can illuminate the discernible dimensions of psychological change without reducing its interpretive complexity. By attending to modality, process type, and cohesive organization as the formal realization of personality and emotion, the study shows how linguistic evidence and psychological theory converge in the analysis of literary narrative.

#### 3.4.5. Summary of findings.

The integration of emotional polarity analysis, SFL-based linguistic interpretation, and personality mapping reveals a coherent trajectory of psychological transformation in *The Garden of Evening Mists*. Yun Ling’s emotional evolution unfolds not through isolated episodes of feeling but through systematic shifts in linguistic form: patterns of restraint, reflection, and reconciliation that index her changing psychological states across the three narrative phases.

In the trauma recollection phase, the predominance of obligation modals, syntactic compression, and affective lexis of withdrawal embodies the protagonist’s conscientious effort to contain grief and sustain moral control. Language here does not express emotion so much as manage it: the recurring negations and paratactic fragments that characterize this phase enact through grammatical form the psychological work of suppression. Yun Ling’s voice in these passages is shaped by what it refuses to say, the rhetorical questions met with monosyllabic negation, the obligation modals turned inward, the affective lexis of withdrawal that defines the trauma register established in the novel’s opening pages.

As the narrative progresses into the Yugiri apprenticeship phase, relational verbs and evaluative lexis of proportion and harmony emerge more frequently, signalling a transition toward aesthetic discipline and cognitive reappraisal. The garden becomes both the literal site and the linguistic medium of this reappraisal: Yun Ling’s growing command of the vocabulary of garden-making, composition, balance, tension, possibility, mirrors her growing capacity to hold psychological complexity without being destabilized by it. Conscientiousness remains high, but Neuroticism begins its decline as the structured practice of aesthetic creation provides a channel for emotions that direct expression cannot contain.

In the reflective closure phase, material and relational processes, permissive modality, and cohesive hypotactic structures mark the culmination of this trajectory. The gradual reduction of affective density corresponds with enhanced emotional regulation and diminishing neurotic intensity, while the sharp rise in Openness markers signals the liberation of a perceptual sensitivity that had always been present but had previously been constrained by grief. Personality transformation in this novel is therefore not merely described through narrative events but enacted linguistically: through evolving modalities, process types, and patterns of cohesion that embody psychological integration at the level of grammar itself.

These findings indicate that emotional language in *The Garden of Evening Mists* functions simultaneously as expression and mechanism: it conveys the protagonist’s inner state while also mediating her adaptation to it. Through language, Yun Ling negotiates the boundaries between silence and disclosure, guilt and forgiveness, restraint and release. More broadly, the study demonstrates that SFL-based qualitative analysis, when integrated with a personality framework, can illuminate the psychological architecture of literary narrative, showing how emotion, cognition, and identity coalesce within the patterned texture of literary form.

## 4. Conclusion

This study examines how personality and emotion are linguistically realized in Tan Twan Eng’s *The Garden of Evening Mists*, drawing on the Big Five Model of Personality (BFM) and Systemic Functional Linguistics (SFL) to construct an integrated theoretical-qualitative framework for the analysis of character language in literary narrative. Through close discourse analysis of selected passages organized across three narrative phases, namely trauma recollection, Yugiri apprenticeship, and reflective closure, the study has traced how patterns of modality, process type, and cohesion correspond to shifts in emotional polarity and their underlying personality dimensions.

The findings indicate that emotional polarity in the novel can be read as a form of psychological adaptation rather than static sentiment. In the trauma recollection phase, negative polarity, realized through obligation modals, syntactic compression, and affective lexis of withdrawal, indexes a high-Conscientiousness, high-Neuroticism personality constellation characteristic of trauma-related self-regulation. As the narrative moves into the Yugiri apprenticeship phase, neutral polarity emerges through evaluative balance and descriptive cohesion, signalling the disciplined reappraisal of emotion through aesthetic practice. In the reflective closure phase, positive polarity becomes dominant, realized through relational processes, permissive modality, and declarative acceptance, corresponding to increased Openness and diminished neurotic tension. These shifts align with observations that personality traits are systematically mirrored in discourse-level language patterns, particularly through modality, evaluative lexis, and cohesive organization.

At the theoretical level, the integration of BFM and SFL suggests that, in this novel, personality is not merely described in language but enacted through it. The protagonist’s progression from restraint to reconciliation exemplifies how emotional regulation and moral identity are linguistically realized through modality shifts, cohesive density, and process variation. The analytical framework developed here, grounded in the correspondence between BFM personality dimensions and SFL metafunctional categories, offers a theoretically coherent and interpretively transparent approach to literary discourse analysis that extends beyond the Western literary canon to engage postcolonial narrative fiction. In this respect, the study responds to a recognized gap in the field: while existing work in personality-oriented text analysis has focused predominantly on non-literary corpora and social media language, the present framework illustrates how BFM-SFL integration can be brought to bear on the stylistically complex language of literary prose, offering a working framework for further investigation.

Several limitations of the present study warrant acknowledgement. The analysis is based on a single novel, and the interpretive associations established between linguistic features and personality dimensions reflect the specific literary context of *The Garden of Evening Mists* rather than generalizable empirical claims. The mapping between emotional polarity and personality traits, particularly the association of negative polarity with the high-Conscientiousness, high-Neuroticism constellation, is context-specific and should not be extrapolated to other texts or genres without further investigation. Single-text, single-analyst qualitative analysis carries inherent risks of interpretive subjectivity. The readings presented here were developed by one analyst through close reading, and although the interpretive categories were checked for consistency across successive readings, the findings should be understood as theoretically informed interpretations rather than independently validated classifications. Readers are accordingly invited to weigh them against the textual evidence presented throughout, which is cited by page number to permit independent scrutiny. The study also acknowledges the well-documented limitations of sentiment and emotion analysis in literary texts, where figurative language, irony, metaphor, and narrative perspective complicate the assignment of affective polarity in ways that resist systematic categorization [34].

Future research could extend this framework to larger literary corpora or cross-linguistic comparisons, exploring how emotional polarity and personality expression vary across cultures, genres, and narrative traditions. Integrating additional personality dimensions, including those offered by the HEXACO model, which provides a more differentiated account of emotionality and moral disposition, may further enrich the analytical framework, particularly for texts in which questions of honesty, humility, and interpersonal ethics are central. Combining the present qualitative approach with corpus-based methods could also address the limitations of generalizability in single-text analysis, providing a more robust empirical foundation for the theoretical mappings proposed here. Ultimately, this study demonstrates that the integration of personality psychology and systemic-functional analysis, when grounded in close reading and theoretical accountability, offers an empirically informed and interpretively coherent framework for understanding the emotional architecture of literary language and the linguistic realization of selfhood in narrative discourse. The dominant linguistic features and personality associations identified across the three narrative phases are summarized in Supplementary S1 Table.

## Supporting information

S1 Table Summary of dominant linguistic features and personality associations across the three narrative phases of *The Garden of Evening Mists.* (DOCX)
