# Supplementary material for: Linguistic realizations of personality and emotional polarity in Tan Twan Eng’s The Garden of Evening Mists: A systemic functional discourse analysis
Source: PLoS One. 2026 Jul 24;21(7):e0354255. doi: 10.1371/journal.pone.0354255 (PMC13399339; doi:10.1371/journal.pone.0354255)
Supplement: S1 Table — (DOCX) [file pone.0354255.s001.docx]

**Supplementary Materials**

**Supplementary Table S1.** Summary of dominant linguistic features and personality associations across narrative phases in *The Garden of Evening Mists*.

| **Narrative Phase** | **Dominant Linguistic Features** | **BFM Personality Association** |
| --- | --- | --- |
| **Trauma Recollection** | Obligation modals (*must*, *cannot*, *never*, *will not*); negation structures; paratactic clause compression; affective lexis of withdrawal and pain (*pain*, *fear*, *dread*, *grief*); mental process verbs of involuntary recollection (*remember*, *see*, *know*) | **High Conscientiousness / High Neuroticism** |
| **Yugiri Apprenticeship** | Evaluative nouns of proportion and harmony (*balance*, *composition*, *stillness*, *possibility*); reflective and epistemic modality; descriptive cohesion; aesthetic and perceptual process verbs; inclusive first-person plurality | **Stable Conscientiousness / Emerging Openness** |
| **Reflective Closure** | Material and relational process verbs (*restore*, *release*, *let go*, *plant*, *touch*); permissive modality (*may*, *can*, *will*); declarative acceptance; cohesive hypotactic structures; evaluative lexis of acceptance and reconciliation (*peace*, *restore*, *accept*) | **High Openness / Reduced Neuroticism** |

**Note.** Dominant linguistic features are identified through SFL-based close reading of selected passages across three narrative phases. Personality associations reflect context-specific analytical judgements grounded in the literary text of *The Garden of Evening Mists* rather than generalizable empirical claims. BFM = Big Five Model of Personality; SFL = Systemic Functional Linguistics.
